# Safety and feasibility of lapatinib for the treatment of a EGFR1/HER-2-positive advanced gastrointestinal stromal tumor in a cat

**DOI:** 10.29374/2527-2179.bjvm001825

**Published:** 2025-05-23

**Authors:** André Gustavo Alves Holanda, Patrick Antônio Sonaglio Civa, Carlos Eduardo Fonseca-Alves, Denner Santos Dos Anjos

**Affiliations:** 1 Departamento de Cirurgia, Universidade de São Paulo, Faculdade de Medicina Veterinária e Zootecnia., São Paulo, SP, Brazil.; 2 Clínica Veterinária Medicalvet, Chapecó, SC, Brazil; 3 Institute of Veterinary Oncology – IOVET, São Paulo, SP, Brazil.; 4 Departamento de Cirurgia Veterinária e Reprodução Animal, Universidade Estadual Paulista, Botucatu, SP, Brazil; 5 VetPrecision Laboratory, Botucatu, SP, Brazil.; 6 Eletro-Onkovet, Franca, SP, Brazil

**Keywords:** cat diseases, precision oncology, targeted therapy, tyrosine kinase inhibitors, felino, oncologia de precisão, terapia alvo, inibidor de tirosina quinase

## Abstract

Gastrointestinal stromal tumors (GISTs) are uncommon mesenchymal tumors in cats that originate from interstitial cells of Cajal (ICC). ICCs are mesenchymal cells found within the muscle layers of the alimentary tract that facilitate communication between the autonomic nervous system and smooth muscles. In this case, lapatinib, a tyrosine kinase inhibitor (TKI) that targets EGFR1 and HER2, was used as part of precision therapy for metastatic GIST in an 8-year-old, 2.7 kg, spayed, female mixed-breed cat, guided by the high expression of these receptors in the tumor. The treatment resulted in partial remission of liver metastases, with a progression-free survival of 171 days and an overall survival of 192 days after starting lapatinib. Lapatinib was well tolerated, with minimal adverse gastrointestinal effects. These findings highlight the potential role of molecular profiling in guiding targeted therapy for feline GISTs and suggest that lapatinib may be a viable treatment option for EGFR1/HER2-positive tumors. Further studies are needed to evaluate the efficacy and safety of TKIs in veterinary oncology, as well as their impact on long-term survival and quality of life in feline patients with GISTs.

## Resumo

Os tumores estromais gastrointestinais (GISTs) são tumores mesenquimais incomuns em gatos, originados a partir das células intersticiais de Cajal (ICC). As ICC são células mesenquimais encontradas dentro das camadas musculares do trato alimentar e desempenham um papel na comunicação entre o sistema nervoso autônomo e o músculo liso. Neste caso, o lapatinibe, um inibidor de tirosina quinase (ITQ), que atua sobre EGFR1 e HER-2, foi utilizado como parte de uma terapia de precisão para um GIST metastático em uma gata, sem raça definida, castrada, de 8 anos de idade, com 2,7 kg. O tratamento foi guiado pela alta expressão desses receptors no tumor. A terapia resultou em remissão parcial das metástases hepáticas, com sobrevida livre de progressão de 171 dias, e sobrevida global de 192 dias, após o início do lapatinibe. A medicação foi bem tolerada, com efeitos adversos gastrointestinais mínimos. Esses achados destacam o potencial papel do perfil molecular na orientação da terapia direcionada para GISTs felinos e sugerem que o lapatinibe pode ser uma opção viável de tratamento para tumores positivos para EGFR1/HER-2. Estudos adicionais são necessários para avaliar a eficácia e a segurança dos ITQs na oncologia veterinária, bem como seu impacto na sobrevida a longo prazo e na qualidade de vida de pacientes felinos com GISTs.

## Introduction

Gastrointestinal stromal tumors (GISTs) are uncommon mesenchymal tumors of the alimentary tract that originate from the interstitial cells of Cajal (Rammohan et al., 2013). GISTs have been misdiagnosed as leiomyosarcomas for many years until immunohistochemistry distinguished between the two entities. GISTs have metastatic potential; therefore, it is important to differentiate them from leiomyosarcomas (which classically have low metastatic potential). GISTs were considered smooth muscle tumors based on their histological characteristics until the identification of KIT (CD117) expression and *c-KIT* mutations (Wu et al., 2019). Genotypically, most GISTs are driven by activating mutations in the tyrosine kinase receptors, KIT and platelet-derived growth factor receptor alpha (PDGFR-α), leading to uncontrolled cell proliferation and tumor growth (Wang et al., 2021).

Surgery is the treatment of choice for GISTs because these tumors are commonly resistant to radiation therapy and conventional chemotherapy (Rammohan et al., 2013). In human medicine, all approved systemic agents for treating GIST are based on orally available tyrosine kinase inhibitors (TKIs), which are small-molecule drugs that induce apoptosis and inhibit cell proliferation (Schaefer et al., 2022; Değirmenci et al., 2022). Imatinib is considered the first-line agent for GISTs with a high risk of recurrence. It inhibits KIT, PDGFRα, and BCR-ABL (Mechahougui et al., 2023). In veterinary medicine, there are no well-established protocols for systemic treatments; however, the use of TKIs has been reported, with descriptions of the use of imatinib (Treggiari et al., 2023) and toceranib (Elliott et al., 2017) in dogs. However, GIST are very rare in cats, and no well-established TKI protocols exist for the systemic treatment of this disease.

Over the past few decades, molecularly targeted therapies for cancer treatment have been developed and refined (Cabanos & Hata, 2021). One example is the immunostaining of EGFR1 (epidermal growth factor receptor-1) and HER-2 (human epidermal growth factor receptor 2), which are considered potential predictors of tumor response to lapatinib (Anjos et al., 2024). Lapatinib is a TKI that binds to adenosine triphosphate (ATP) sites within the intracellular tyrosine kinase domains of cancer cells, thereby inhibiting EGFR1 and HER-2 phosphorylation. Consequently, the MAPK and PI3K signaling pathways are downregulated, leading to cell death (Değirmenci et al., 2022). A previous study demonstrated the therapeutic potential of lapatinib in combination with piroxicam in dogs with HER2-positive muscle-invasive urothelial carcinoma. Compared to dogs treated with piroxicam alone, lapatinib-treated dogs showed a greater reduction in primary tumor size and improved survival (Maeda et al., 2022).

Lapatinib has been studied in cats following a single dose administration (Yu et al., 2024). After a single dose of 25 mg/kg, cats did not present with side effects, and lower serum bioavailability was observed in cats than in dogs (Yu et al., 2024). However, these authors did not evaluate the effects of long-term administration or antitumor effects. Therefore, in the present case, we report the clinical outcomes of a cat with metastatic GIST treated with lapatinib guided by a precision medicine approach.

## Case description

A case report of the diagnosis and initial approach for this patient (an 8-year-old, 2.7 kg, spayed female, mixed-breed cat) was previously published (Civa et al., 2024) because of its novelty. At the time of publication of this case report, the patient had been followed up for 485 days. The patient was diagnosed with GIST based on morphological and immunohistochemical findings. Histopathological analysis revealed elongated, spindle-shaped neoplastic cells with poorly defined margins originating from the muscular layers and a mitotic index of 2/10 per high-power field (hpf) (400× magnification). Immunohistochemical analysis for S100, Desmin, 1A4, and HHF35 was negative, whereas that for KIT was positive. The tumor sample was subjected to a multikinase® panel and revealed HER-2 (human epidermal growth factor receptor 2) (65%) and EGFR1 (epidermal growth factor receptor-1) (50%) overexpression, VEGFR-2 (vascular endothelial growth factor receptor 2) (35%), PDGFR-beta (platelet-derived growth factor receptor beta) (15%), and mild c-KIT expression (15%).

Toceranib (Palladia®) was prescribed at a dose of 2.75 mg/kg orally, following a schedule of three days per week (Monday, Wednesday, and Friday). The medication was administered for 10 weeks without any adverse effects. However, during patient follow-up, tumor progression was identified based on the identification of presumed liver metastases and increased tumor volume (35% of the basal evaluation), and the treatment with toceranib was discontinued.

Based on the high dual expression of EGFR-1 and HER-2, treatment with compounded oral lapatinib (Tykerb®) was proposed. As no previous reports demonstrating the safety of lapatinib were published when we started treatment, we started with lower doses (compared to dog treatment) and progressively increased them. The dose was gradually increased according to the tolerance of the patient. Initially, lapatinib was prescribed at 2.5 mg/kg once daily for 15 days. The dose was then increased to 5 mg/kg once daily for 20 days and subsequently to 7.5 mg/kg once daily for an additional 20 days, with no adverse effects observed. The dose was adjusted and maintained at 10 mg/kg daily until further recommendations were provided.

**Figure 1:**
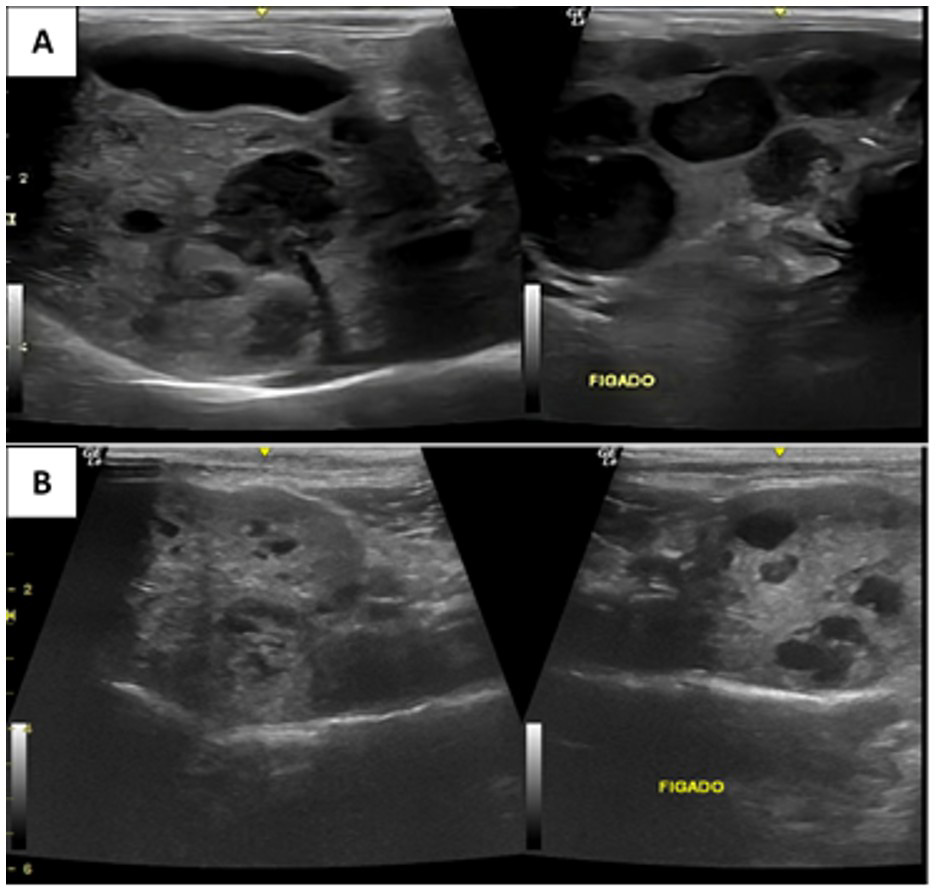
Ultrasonographic assessment of GIST liver metastasis in a cat, highlighting hypoechoic lesions. (A) Before treatment with lapatinib and (B) after 45 days of treatment with lapatinib, a partial response was observed.

The first assessment of the treatment response was conducted 45 days after initiating lapatinib, revealing a partial response of the liver metastases (Figure 1) based on the Veterinary Cooperative Oncology Group (VCOG) guidelines (Nguyen et al., 2015). The follow-up examination intervals were adjusted based on the owners’ financial constraints. The cats were monitored for blood counts and renal and liver profiles every 60 days and abdominal ultrasonography and thoracic radiography every three months. The only adverse effects observed during treatment were three episodes of vomiting and diarrhea, which did not require medical intervention or discontinuation of medication and were classified as grade 1 (LeBlanc et al., 2021).The patient achieved a progression-free survival (PFS) of 171 days after the initiation of lapatinib.

After 171 days of treatment, new nodules were detected in the hepatic parenchyma, indicating the presence of metastatic lesions. Blood test results were within the reference range, except for hypoalbuminemia (2 g/dL; RI 2.1–3.3 g/dL). The animal’s clinical condition progressively worsened, with anorexia, loss of muscle mass, dyspnea, tachypnea, respiratory failure, and death after 21 days, resulting in 192 days of survival after lapatinib treatment. The owner did not consent to a necropsy, and pulmonary metastasis was suspected. The patient achieved 656 overall survival (OS) days based on the proposed personalized approach.

## Discussion

Part of this case was previously published owing to its novelty (Civa et al., 2024), and this case report focused only on the use of lapatinib for treatment. Lapatinib treatment was not used in the previous case report because the owner decided whether to accept the treatment until the publication of the case report. The present study demonstrated that the use of lapatinib in a feline with advanced GIST positive for EGFR1 and HER-2 resulted in a partial response to liver metastasis, with a PFS of 171 days and an OS of 192 days after starting lapatinib. Interestingly, these patient had aggressive GIST, with an OS of 656 days. Based on the current knowledge regarding the use of TKI inhibitors for treating GIST in dogs and humans, lapatinib has not been proposed for patient treatment. In this case, lapatinib was administered based on HER-2/EGFR1 expression. These findings reinforce the importance of understanding the molecular profile of tumors using a tyrosine kinase receptor panel. Thus, tumor expression can be systematically analyzed to predict patient responses to targeted therapy.

A significant number of GISTs exhibit activating mutations in the *KIT* and *PDGFRα* genes; however, variations in this pattern may occur. In humans, the most commonly identified driver mutations in GISTs involve *KIT* (60–70%) and *PDGFRα* (10–15%) mutations. Nonetheless, approximately 10–15% of GIST cases lack mutations in these genes and are classified as “wild-type GISTs.” These tumors typically do not respond to imatinib, the standard treatment for GISTs with *KIT* or *PDGFRα* mutations (Nishida et al., 2024). Similarly, Fujii et al. (2022) reported a cat with wild-type GIST that was unresponsive to treatment with imatinib. The authors emphasize the need for further evaluation to enable the individualized treatment of patients.

In our multi-kinase ^®^ panel, the tumors exhibited overexpression of HER-2 (65%) and EGFR1 (50%) and reduced expression of VEGFR-2 (35%), PDGFR-beta (15%), and c-KIT (15%). Initially, two possible TKIs were considered: toceranib, a tyrosine kinase inhibitor that targets VEGFR-2, PDGFR-β, and c-KIT, and lapatinib, an inhibitor of EGFR1 and HER-2. Owing to the lack of previous clinical studies on lapatinib in feline species, the use of toceranib has been proposed, despite reduced tumor expression of its therapeutic targets. Toceranib is used off-label in cats, with reports of its safe use and clinical benefits in the treatment of oral squamous cell carcinoma (Olmsted et al., 2017) and mast cell tumors (Berger et al., 2018). Although toceranib was well tolerated by the patient, liver metastasis progressed within 10 weeks, possibly because of the low expression of therapeutic targets for the medication, which was subsequently discontinued and replaced with lapatinib.

In human oncology, immunohistochemistry is widely used as a predictive marker for cancer treatment (Mino-Kenudson, 2017; Zhao et al., 2020). However, research in veterinary medicine is still in its infancy. Recently, the expression of HER2, EGFR1, VEGFR-2, PDGFR-β, c-KIT, and ERK1/ERK2 has been investigated in various canine tumors, with mesenchymal tumors showing the lowest expression levels and carcinomas showing the highest expression levels. The authors emphasized the importance of exploring whether tumors from patients expressing more than 50% of these receptor targets would show a better clinical response to targeted therapy than those that do not (Anjos et al., 2024). In our case, the patient’s GIST liver metastasis responded to treatment with lapatinib, which targets the receptors with the highest expression in the tumor (HER2, 65%; EGFR1, 50%). Despite their initial efficacy, the disease progressed after 171 days of treatment, reflecting the possibility that targeted therapies may eventually fail in advanced cancers as tumors develop resistance (Vander Velde et al., 2020).

Mutations in *EGFR1* have been reported in less than 1% of human GIST cases and are commonly associated with a low risk of metastasis and recurrence (Shi et al., 2017). In contrast, HER-2 expression has been described in 43.7% of cases and is considered an independent prognostic factor, as it is correlated with an increased risk grade of GIST, tumor size, mitotic count, and tumor relapse (Abd El-Aziz et al., 2017). No studies have investigated the prevalence of EGFR1 or HER-2 expression in patients with GIST in the field of veterinary medicine. However, HER-2 overexpression has been observed in other types of cancers in animals, such as urothelial carcinoma in dogs, for which lapatinib has demonstrated therapeutic efficacy. The treatment is considered well-tolerated, with adverse effects including elevated liver enzymes, vomiting, diarrhea, hyperpigmentation, pruritus, and alopecia (Maeda et al., 2022).

To the best of our knowledge, no previous study has used lapatinib to treat tumors in cats. A recent pharmacokinetic study demonstrated that a single oral administration of lapatinib at a dose of 25 mg/kg resulted in a lower average maximum plasma concentration and shorter half-life in cats than in dogs. Thus, a higher dose or shorter dosing interval may be recommended for cats to achieve plasma concentrations similar to those in dogs. However, feline species may exhibit greater variability among patients (Yu et al., 2024). In the present case, we gradually increased the daily lapatinib dose. The dose was stabilized at 10 mg/kg because of the occurrence of mild gastrointestinal adverse effects (vomiting and diarrhea), which did not require discontinuation of the medication. At this point, it is impossible to rule out the possibility that gastrointestinal signs were related to liver metastases.

## Conclusions

Lapatinib may represent a promising and safe systemic therapy for cats with GIST overexpressing EGFR1/HER-2. Multi-kinase panels appear to be a viable option for predicting responses to targeted therapies, allowing personalized treatment. Future studies should assess these findings using a larger number of animals to confirm these findings.
